# A daily peat moisture code for monitoring and predicting peatland fire danger

**DOI:** 10.1016/j.mex.2026.104174

**Published:** 2026-09-23

**Authors:** S.L. Wilkinson, G.J. Verkaik, D.K. Thompson, J.M. Waddington, E. Arango Ruda, B.M. Wotton

**Affiliations:** a School of Resource and Environmental Management, Simon Fraser University, Burnaby, BC, V5A 1S6, Canada; b School of Earth, Environment and Society, McMaster University, Hamilton, ON, L8S 4K1, Canada; c Northern Forestry Centre, Canadian Forest Service, Natural Resources Canada, Edmonton, AB, T6H 3S5, Canada; d Great Lakes Forestry Centre, Canadian Forest Service, Natural Resources Canada, Sault Ste. Marie, ON, P6A 2E5, Canada; e Institute of Forestry and Conservation, University of Toronto, Toronto, ON, M5S 3B3, Canada

**Keywords:** Peat, Fire, Risk

## Abstract

Peatlands, ecosystems with deep (>40 cm) organic soils, play diverging roles in landscape wildfire regimes based on their moisture status. When peatlands are dry they can be the source of high-severity fire and carbon and smoke emissions. The daily Peat Moisture Code (PMC) presented here calculates the change in storage in the peatland water balance and accounts for peatland ecohydrological feedbacks by integrating peat hydrophysical properties using novel equations. Evapotranspiration is modelled using a modified Priestley-Taylor method. The change in water storage is converted to an index (the PMC) that correlates with water table position by accounting for the Specific Yield of peat at different depths and a negative (dampening) feedback on evapotranspiration. The PMC represents the moisture status of peatlands where high PMC values indicate dry fuel conditions driving higher fire danger and high-severity burns. Exemplary fire danger thresholds are presented for the example to assist with interpretation. The PMC is a generic method for quantifying peatland fire danger in varying ecoregions and peatland types.

● Water balance model with daily bookkeeping approach

● Minimal data requirements consistent with federal and provincial weather stations

● Represents the methodological basis for the hourly PMC of the next-generation Canadian Forest Fire Danger Rating System

## Graphical abstract

**Figure:**
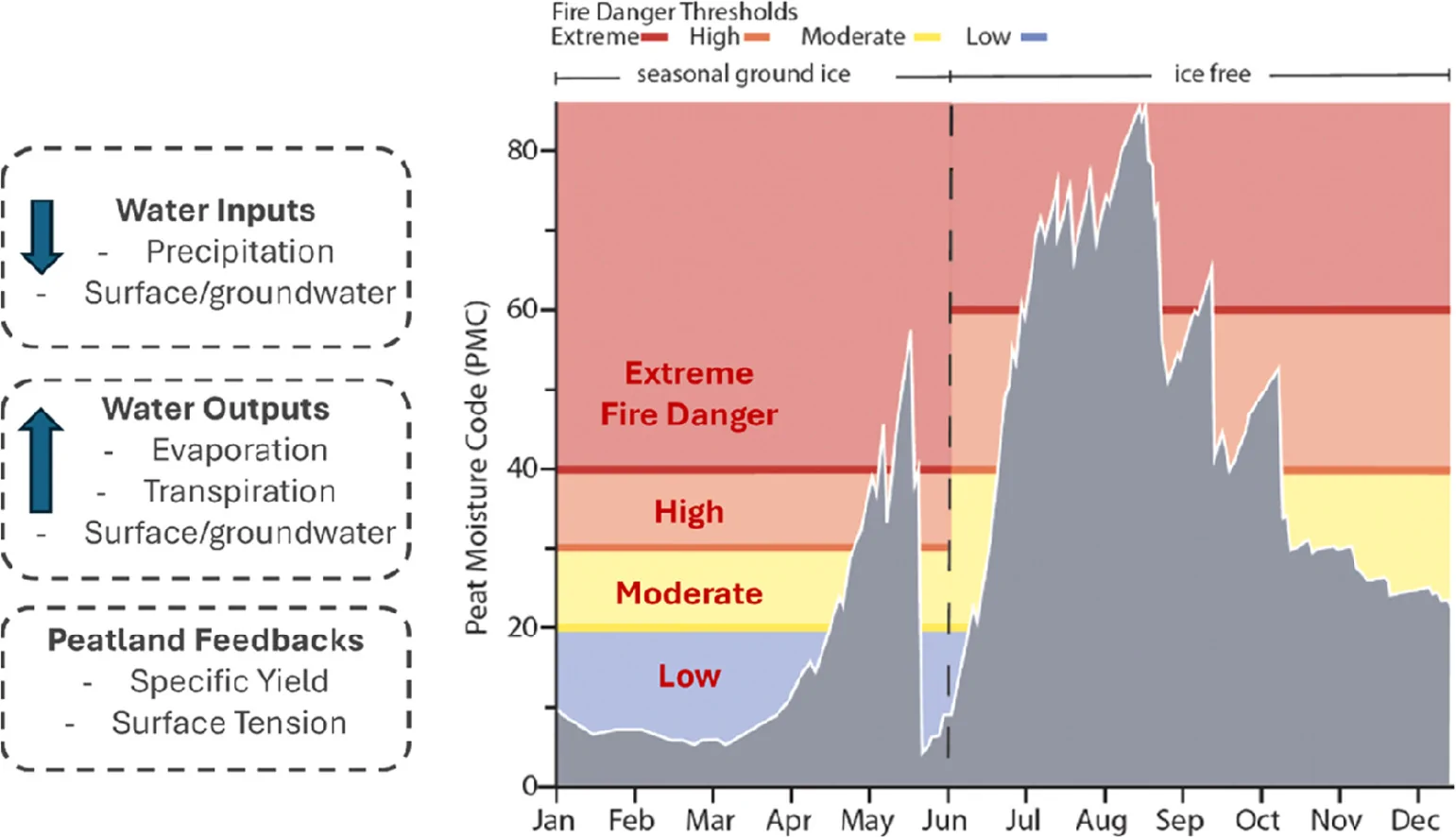

Specifications table

**Table:** 

**Subject area**	Environmental Science
**More specific subject area**	Wildfire Science and Management
**Name of your method**	Daily Peat Moisture Code
**Name and reference of original method**	NA
**Resource availability**	Related R code is available in: (https://github.com/ElizabethArangoRuda/PMC)

## Background

The development of a daily Peat Moisture Code (PMC) follows the identification of a need for modelling fuel moisture in ecosystems with deep organic soils, as their moisture response differs significantly from the standard pine fuel type on which other fuel moisture codes were developed [1,2]. The daily PMC presented here will serve as the methodological basis for the hourly PMC that is to be incorporated into the next-generation Canadian Forest Fire Danger Rating System (CFFDRS) FWI2025 [3].

Peatlands, wetlands with deep (>40 cm) peat or organic soil, cover 12% of Canada’s land area (Fig. 1) and up to 40% in some ecoregions [4,5]. While they can sometimes act as fire refugia (areas that avoid burning; [6]), they are generally well integrated into regional wildfire regimes [[7], [8], [9]]. Increasing peatland cover does not correlate with lower regional burn rates, further indicating that the peatland presence does not control landscape-level fire dynamics [10]. Canada and many other regions (e.g., Europe, Siberia, China) are now frequently experiencing long-lasting fires that are difficult to suppress, extensive smoke production and carbon emissions, and new or more overwintering fires, all of which are associated with peatland fires [11,12]. Due to the unique hydrophysical structure of peatlands compared to upland forests, and the complex hydrological feedbacks acting within them [13], the PMC has been specifically developed to estimate, track, and predict fire danger in peatlands. The method described here is parameterised for the most hydrologically simple peatland type in Canada, the treed bog.

**Fig. 1:**
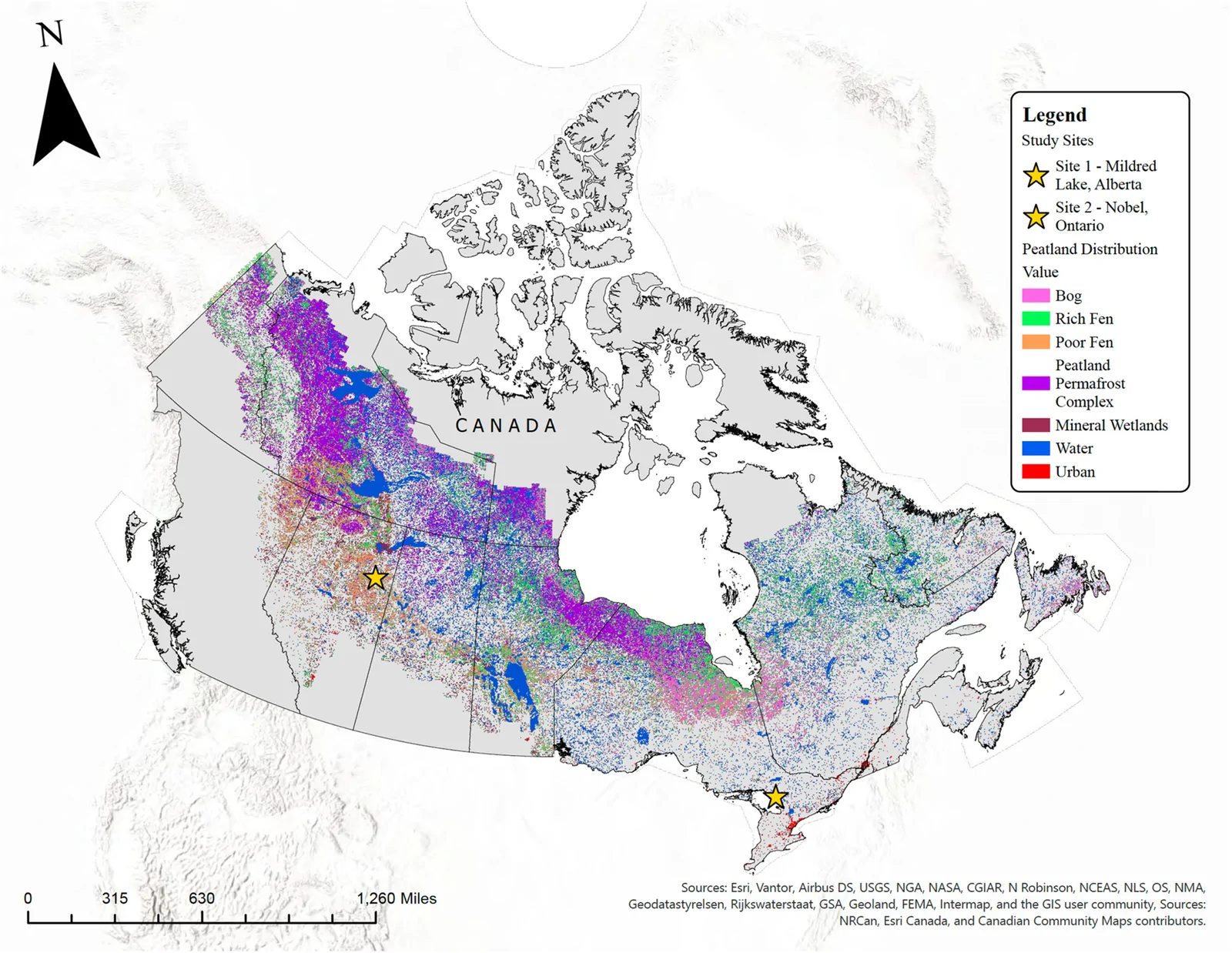
Map of peatland subtypes in boreal Canada. Yellow stars show the location of the two sites used for testing and development of the daily Peat Moisture Code. Data is sourced from Pontone, N., Millard, K., Thompson, D.K., Guindon, L. and Beaudoin, A. (2024), A hierarchical, multi-sensor framework for peatland sub-class and vegetation mapping throughout the Canadian boreal forest. Remote Sens Ecol Conserv. https://doi.org/10.1002/rse2.384.

The PMC has been designed akin to a simplified peatland water balance model (Fig. 2) to represent the ignition and combustion potential of below-ground peat and above-ground peatland vegetation, including the potential for high-severity smouldering combustion. This water balance model uses a bookkeeping approach, whereby calculations of moisture change are applied to the previous day’s value to arrive at the current day’s value, which is similar in design to the Drought Code (DC) and Duff Moisture Code (DMC) components of the Fire Weather Index (FWI) system in the CFFDRS. The PMC represents storage in the peatland water balance (i.e., moisture content) and is calculated by accounting for precipitation, evapotranspiration, surface/groundwater inputs, and peat hydrophysical properties. The PMC can be conceptualized as an “effective” water table depth (WTD) in cm, calculated as a positive value; the larger the value, the deeper the water table (WT) is expected to be. Therefore, the PMC increases with decreasing water storage (moisture content), similar to other moisture codes in the FWI System. It is restricted to small negative values at the lower end, representing saturated peat conditions and very low fire potential, and is unbounded at the upper end. Here, we present a generic PMC; however, specific modifications and interpretation of the PMC for the range of peatland types and ecozones across the country should be made based on historical distributions of PMC for each ecozone, and if WTD and peat property data are available, for varying peatland types.

**Fig. 2:**
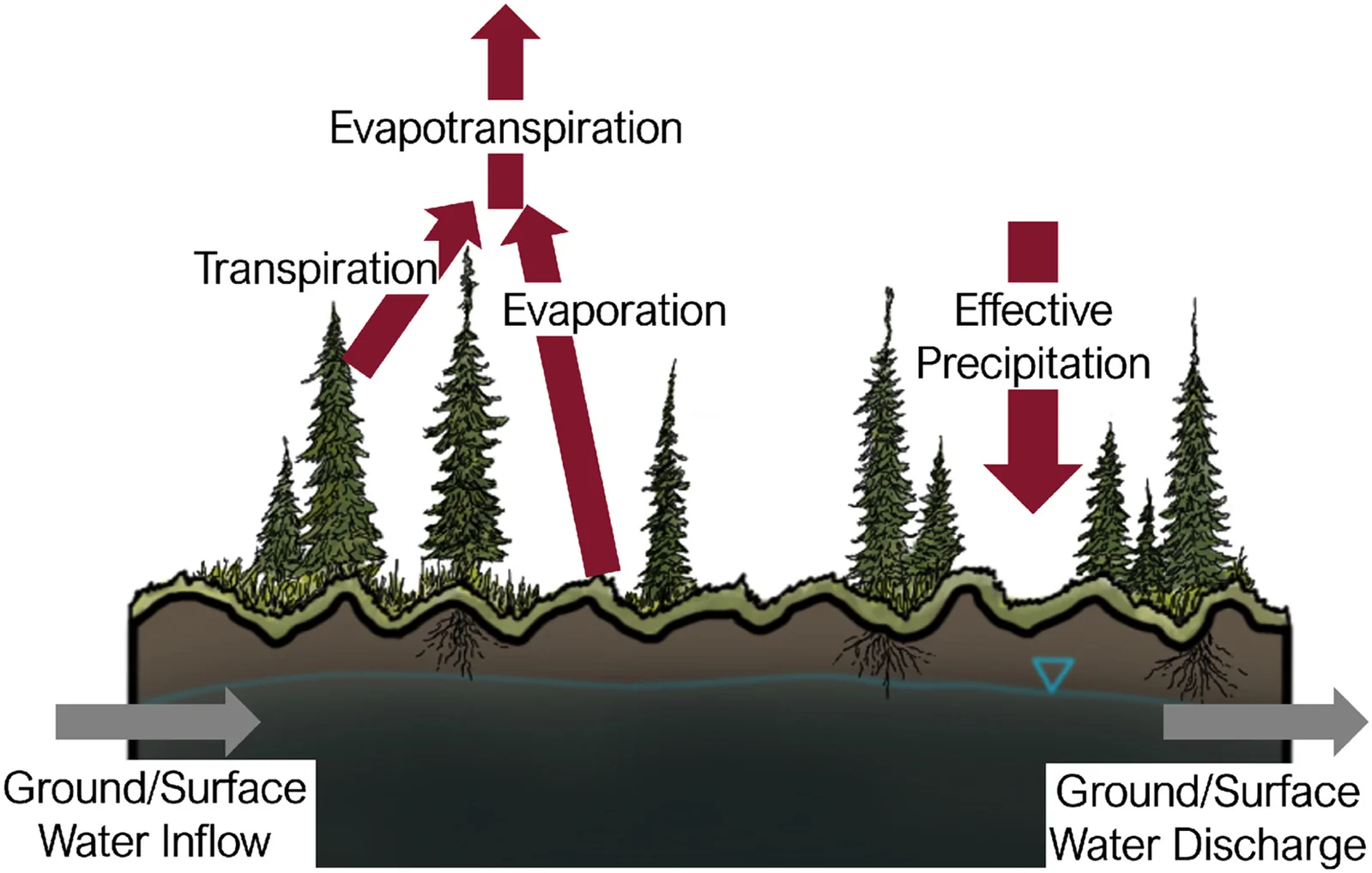
Inputs: Precipitation (Effective precipitation = Precipitation – Interception). Outputs: Evaporation from surface, Evapotranspiration from leaves/needles. Optional: Input from groundwater or surface water, Output from surface water or groundwater recharge; these are currently set to zero but can be added through code modification.

## Method details

### Peatland water balance model

A water balance model is an approach used to quantify and keep track of the inputs and outputs of water in a system. Taking this approach to a peatland ecosystem means accounting for the typical hydrophysical properties of peat (highly organic soil) that are specific to peatlands. In Canada, peatlands are defined as containing at least 40 cm of peat above mineral soil or bedrock [14]. The properties of this peat, and therefore the interactions with water inputs and outputs, tend to vary with depth. Hence, in this model, we use empirical data on peat properties (with depth) to more accurately track changes in storage. The inputs and outputs of water included in the PMC model are shown in Fig. 2. Evaporation represents the loss of water from the ground (peat) surface, transpiration is the loss of water due to above-ground vegetation, and evapotranspiration is the combined effect of these processes. Effective precipitation is calculated as the precipitation amount minus the amount of precipitation intercepted by the tree canopy (therefore not reaching the ground). Groundwater and surface water inputs are constant inputs that can be associated with rain events or set to zero, as is the case in our simple example for a treed bog.

The PMC operates on a long (up to multi-decadal) timescale to account for potential multi-year water deficits in peatland moisture status and the long fuel time-lag of peat, i.e., slow drying and rewetting processes. The PMC is designed to approximate peatland WTD and, therefore, the overall moisture status of peatland fuels. Hence, there is no strict upper bound on the PMC, although increases in fire danger and smouldering potential will diminish with ever-increasing code values. Daily meteorological parameters will serve as the inputs for driving the PMC model, and PMC output values will be interpreted with respect to thresholds to varying levels of fire danger (see Fire Danger Threshold section). Each component of the PMC calculation is configurable to enable parameterisation to a range of peatland types (e.g., open, shrubby, treed; bog, fen, swamp). Default parameter suites for this broad range of Canadian peatland types will be developed as the daily PMC is used across a variety of ecozones. The general form of the PMC presented here, using daily data, is based on a hydrologically simple and fire-prone peatland type in Canada, a treed bog (i.e., a peatland with limited groundwater and surface water connectivity and an established coniferous tree cover; see Fig. 3; Table 1). The PMC estimates both below- and above-ground fire potential. Thus, the estimated below-ground fuel consumption, via the sustained smouldering combustion of peat, and the expected fireline intensity (energy output at the fire front) will increase as the PMC increases.

**Fig. 3:**
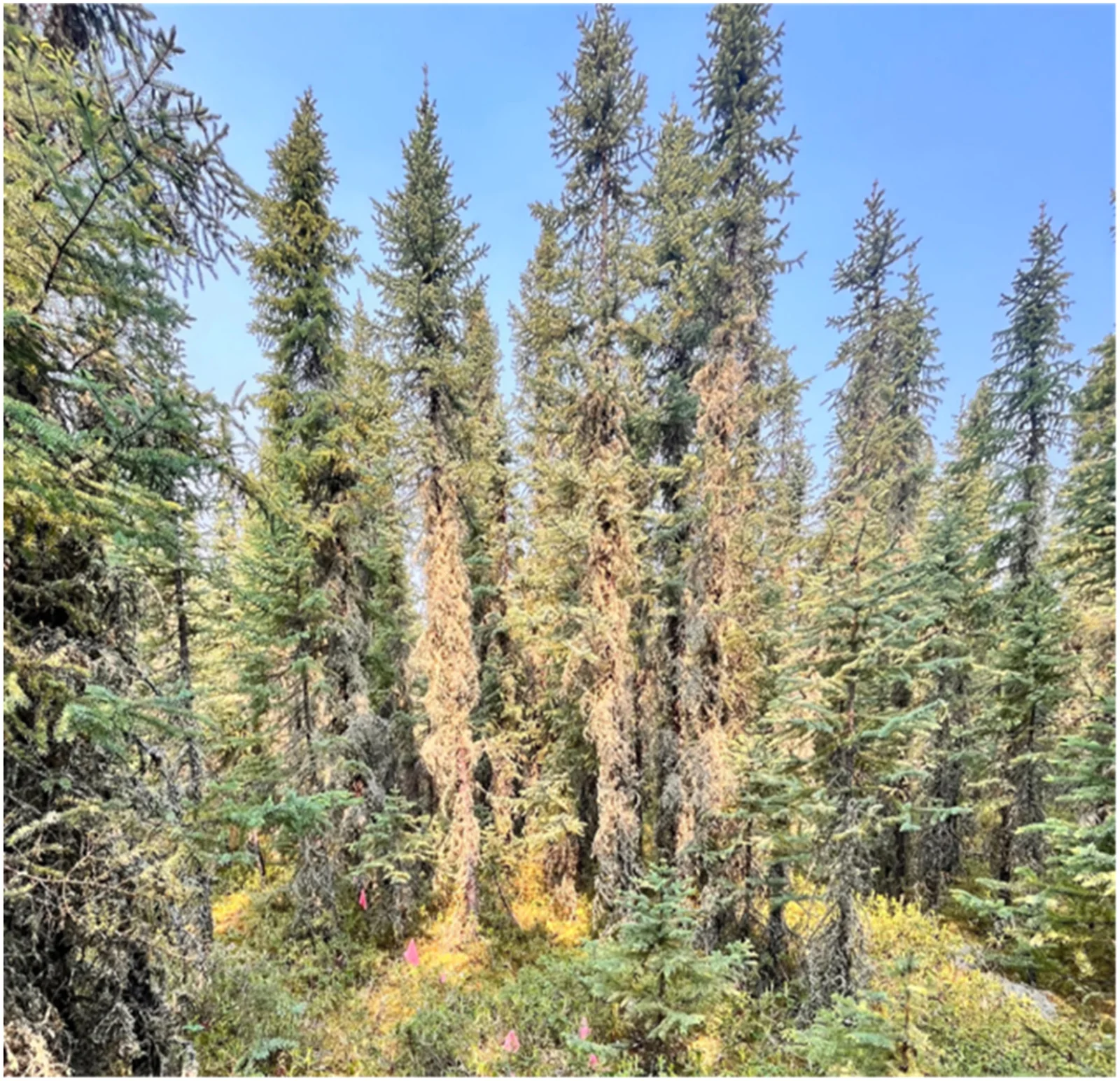
Treed bog; black spruce dominated with ample surface and ladder fuels and a groundcover of Sphagnum or feather mosses. This is the “default” peatland type used to exemplify the daily PMC. Photo credit: Adam Scott.

**Table 1:** Treed Bog characteristics and description of wildfire fuels present (Alberta Environment and Sustainable Resource Development, 2015 and other sources).

Peatland Class Characteristics	Wildfire Fuel Description	Typical Fuel Load
Peatland; more than 40 cm of peat, often more than 1 or 2 m, infrequently up to 8 m	Significant below-ground fuel strata made up of peat (organic soil) typically increase in bulk density (and carbon content) with depth.	0.122 g cm^-3^ (ranges from 0.068 to 0.176 g cm^3^) [15]
Predominantly fed by precipitation (as opposed to surface and groundwater), driving low salinity levels (less than 100 µS/cm conductivity)	Below-ground, surface and above-ground fuel strata are sensitive to climatic and weather patterns such as periods of high temperatures, low humidity and drought.	
Ground cover dominated by mosses (*Sphagnum* or feather mosses) and porewater pH of less than 5.5	Mosses do not have vascular roots (they are bryophytes), meaning they cannot access deep water stores and can dry out easily based on weather conditions. Mosses die and (slowly) decompose, creating the peat on which they sit.	0.1-0.30 kg m^-2^ (*Sphagnum*) [16] 0.05-0.23 kg m^-2^ (feather moss) [17]
Dominant surface cover of ericaceous shrubs such as Common Labrador Tea (*Rhododendron groenlandicum*)	Woody shrubs contribute to the surface (understory) fuel load and can act as ladder fuels, connecting the surface and (larger) canopy fuel loads.	0.10-1 kg m^-2^ [16,18]
More than 25% tree cover (primarily Black Spruce; *Picea mariana*)	Coniferous trees, such as Black Spruce, have needles with a lower water content than green leaves. Needles, fine branches, branches and bark make up the majority of fuel available for combustion during wildfire. These are collectively termed canopy fuels.	0.80-1.4 kg m^-2^ (Canopy fuel load) [[18], [19], [20], [21]]

Because the PMC represents the water storage in peatlands by approximating effective WTD, a number of studies were used to suggest some generalised thresholds for different categories of fire danger (see Application and Interpretation section). Generally, as WTD increases (peatland water storage decreases), fire severity increases. This is evidenced by a number of post-hoc peatland fire severity studies in undisturbed peatlands [[22], [23], [24]] in anthropogenically-drained peatlands where the WTD was purposefully increased [19,[25], [26], [27]], and in physically-based and statistical modelling studies [[28], [29], [30]].

## Data requirements

The daily PMC is calculated using daily fire weather data inputs. The specific data requirements vary depending on the data available, as the calculation has been designed to work effectively with standard on-site, local, regional, or modelled weather data. The data requirements of the daily PMC are as minimal as possible to support long-range back-calculation and use in remote, data-limited regions, and to be compatible with the data available from current FWI weather monitoring stations across Canada (Tables 2 and 3). The hourly PMC, as part of the updated FWI2025, will be calculated hourly, resulting in a modified methodology.

**Table 2:** Data requirements (inputs) for the daily Peat Moisture Code calculation, including the modelling of evapotranspiration.

Variable Name	Units	Model Component	Notes
Daily Precipitation (P)	Daily sum in cm	Water Input	Combined rain and snow precipitation data is preferred
Daily Potential Evapotranspiration (PET)	Daily sum in cm	Water Output	Can be calculated using the Priestley-Taylor method as described below. Other calculations can be used depending on data availability (i.e., Penman/Penman-Monteith), as direct measurement of AET.
Daily Maximum Temperature (T_max_)	From measured hourly data, daily average or modelled data in degrees Celsius	Modelled PET	
Daily Minimum Temperature (T_min_)	From measured hourly data, daily average or modelled data in degrees Celsius	Modelled PET	
Daily Average Temperature (T_avg_)	From measured hourly data, daily average or modelled data in degrees Celsius	Modelled PET	Optional, can be calculated from T_max_ and T_min_
Ground/Surface Water (GW)	Daily sum in cm of water depth equivalent	Water Input Water Output	Can be provided once as input and once as output, or as a net change in storage.

**Table 3:** Parameters for calculating the Daily Peat Moisture Code.

Parameter	Unit	Model Component	Configurable by user	Notes
Peat Specific Yield (Sy)	Value between 0 and 1	PMC Output	Yes, but not required	Value changes with PMC value (i.e., water table depth (WTD)). A depth profile for the general peatland type is provided as the default. The default minimum value is 0.1.
Day of Year	Julian day (1-366)	Modelled PET	NA	
Latitude	Decimal Degrees North	Modelled PET	Yes, required if using extraterrestrial radiation (K_ext_) calculation.	Conversion to radians provided.
Interception	cm of water	Water Input	Yes, but not required.	Can be a flat rate across all daily precipitation totals, or can be threshold-based, e.g., only remove rainfall from events < 1mm.
Potential to Actual Evapotranspiration Conversion (PET_multiplier_)	Value between 1 and 0	Water Output	Yes, but not required.	Value changes with PMC value (i.e., WTD depth). A depth profile for the general peatland type is provided as the default. The default minimum value is 0.15.

## Model calculations

### Potential evapotranspiration (PET)

Evapotranspiration represents the removal of water from the system to the atmosphere in the PMC. Firstly, potential evapotranspiration (PET) is required (unless using a direct measurement of AET). PET can be directly entered into the PMC equation if it is being measured on-site (*e.g.*, via an on-site meteorological station with measured radiation balance). For regions relying on standard fire weather station data that lacks net radiation measurements, either measured or modelled, PET can be calculated via the Priestley-Taylor equation using daily minimum and maximum temperature or daily average temperature, along with location data to estimate net radiation at the surface (Table 4; based on Allen et al., [31]).

**Table 4:** Constants in the Priestley-Taylor equation for modelling potential evapotranspiration.

Variable Symbol	Description
ɸ	Latitude in decimal degrees north
μ	Constant to convert latitude in decimal degrees north to radians. Value of 0.0174533 (1° × π/180 = 0.0174533 rad).
α	Priestly-Taylor alpha coefficient. Values range from 1 to 1.26 as the open-water proportion of a wetland system increases [32]. A value of 1 is used here to represent a treed bog with minimal ponded surface water.
γ	Psychrometric constant in kPa°C^-1^. This can be calculated based on input variables or set to a typical value of 0.063 kPa°C^-1^.
G_sc_	Solar constant set at 0.0820 MJ m^-2^ min^-1^
a	Constant in the Hargreaves calculation of Rn from K_ext_. Value of 0.17 used in example.
b	Constant in Hargreaves calculation of Rn from K_ext_. Value of 0.5 used in the example.
λ	The latent heat of vaporization of water is constant at 2453 MJ m^-3^

Priestley-Taylor Equation

Extraterrestrial Radiation (K_ext_)

Measured in MJ m^-2^ day^-1^

(1) $$Declination=0.409*sin\left(\left(2*\frac{\pi }{365}\right)*DOY-1.39\right)$$

(2) $$S_{angle}=\;arccos\left(-tan\;\left(\phi *\;\mu \right)\;*tan\left(Declination\right)\right)$$

(3) $$IRD=1+0.033*cos\left(\left(\frac{2\pi }{365}\right)*\;DOY\right)$$

(4) $$K_{ext}=\frac{24*60}{\pi }*G_{sc}*IRD*\left[S_{angle}*sin\left(\phi *\mu \right)\;*sin\left(Declination\right)\;+cos\left(\phi *\mu \right)\;*cos\left(Declination\right)\;*sin\left(S_{angle}\right)\right]$$

Where *Declination* is the solar declination, *DOY* is the Julian day of year, *S_angle_* is the sunset hour angle, *IRD* is the inverse relative distance between the Earth and the Sun, and *K_ext_* is the Extraterrestrial Radiation measured in MJ m^-2^ day^-1^.

Downwelling Shortwave Radiation (S_w_)

Measured in MJ m^-2^ day^-1^

Where not otherwise recorded, downwelling shortwave radiation (*S_w_*) can be calculated using a variation of the Hargreaves model (Eq. 5).

(5) $$S_{w}=a*\;K_{ext}*{\left(T_{max}-T_{min}\right)}^{b}$$

Net Radiation (Rn)

Measured in MJ m^-2^ day^-1^

(6) $$R_{n}=\;S_{w}*0.72$$

*R_n_* is estimated as a proportion of downwelling shortwave radiation to account for reflection and net longwave radiation (9 years of half-hourly daytime data from a peatland complex near Nobel, Ontario; Unpublished data).

Priestly-Taylor Potential Evapotranspiration (PET)

Temperature (°C)

(7) $$T=\frac{\left(T_{min}+T_{max}\right)}{2}$$

An alternative to the above is to input the daily average temperature directly into the equation.

Slope of the saturation vapour pressure curve (kPa°C^-1^)

(8) $$Slope=0.61365*\left(\frac{17.502}{240.97+T}-17.502*\frac{T}{{\left(240.97+T\right)}^{2}}\right)*e^{17.052*\frac{T}{\;240.97+T}}$$

Potential Evapotranspiration (PET; cm day^-1^)

(9) $$PET=\;\alpha *\frac{Slope}{\left(Slope+\gamma \right)*\lambda }*Rn*100$$

PET is calculated daily; the scaling factor of 100 is used above to convert m day^-1^ to cm day^-1^ (Fig. 4).

**Fig. 4:**
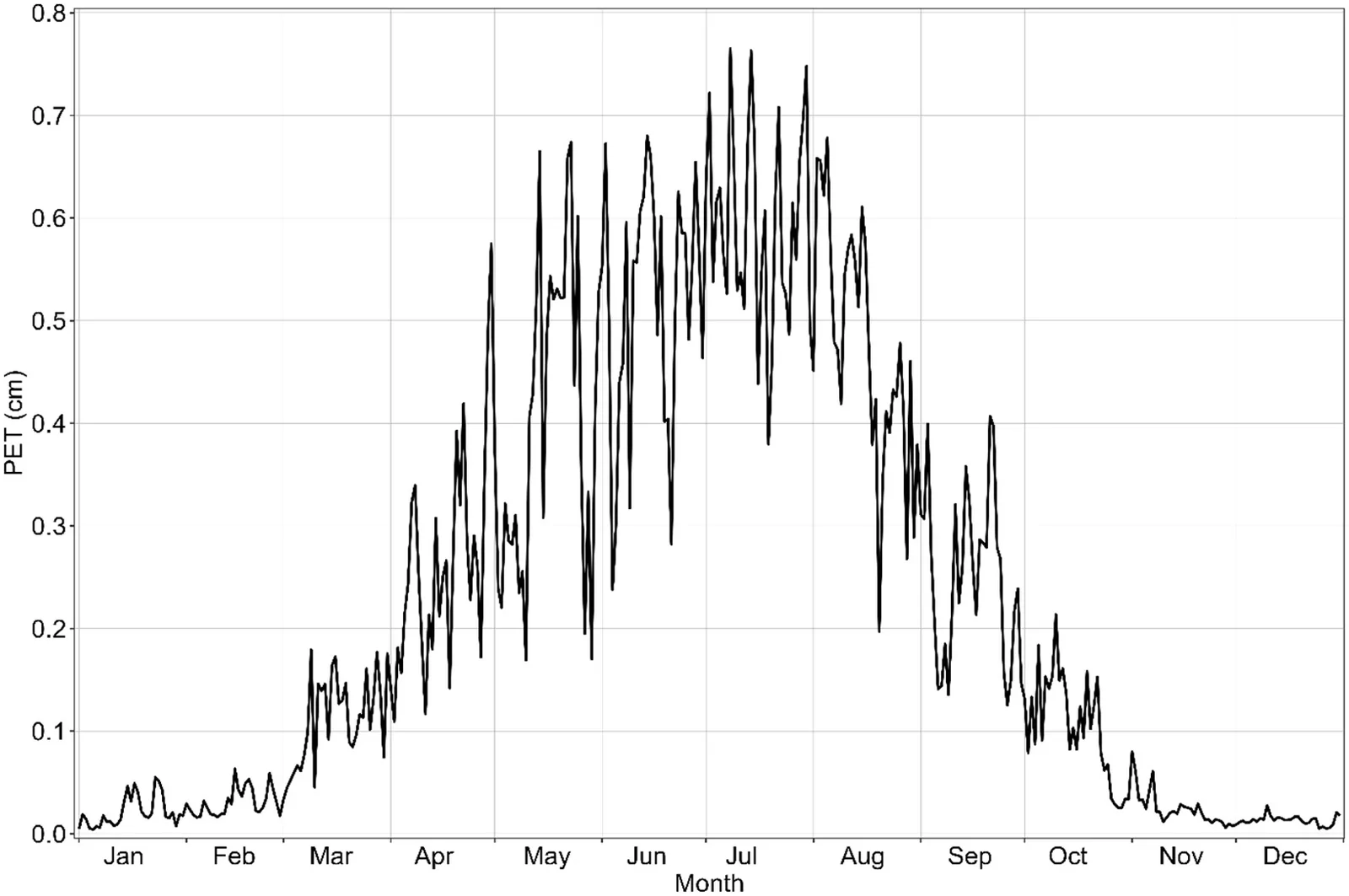
PET calculated using the Priestley-Taylor method described above. Figure uses weather data from the Mildred Lake, AB Environment and Climate Change Canada weather station, 40 km north of Fort McMurray, AB.

### Actual evapotranspiration (AET)

Actual evapotranspiration (AET; cm day^-1^) is derived from PET (above) after accounting for the change in surface resistance of surface-covering mosses. Surface resistance here is used similarly to its use in plant ecophysiology (*i.e.*, to describe the resistance of vapour flow through stomata openings, total leaf area, and soil surface; [31]). However, unlike vascular plants, mosses lack root structures and rely on capillary rise to supply water to the surface. Despite the lack of stomatal control on ET, laboratory and field experiments have parameterised the reduction in ET from the peat surface in relation to the near-surface pore water tension [33]. From this, near-surface tension was related to WTD (Fig. 5, Eq. 10).

(10) $$\text{Equation}\;\text{of}\;\text{the}\;\text{line}:\;y\;=\;2E-08x^{6.4018}\;\;r^{2}\;=\;0.75$$

**Fig. 5:**
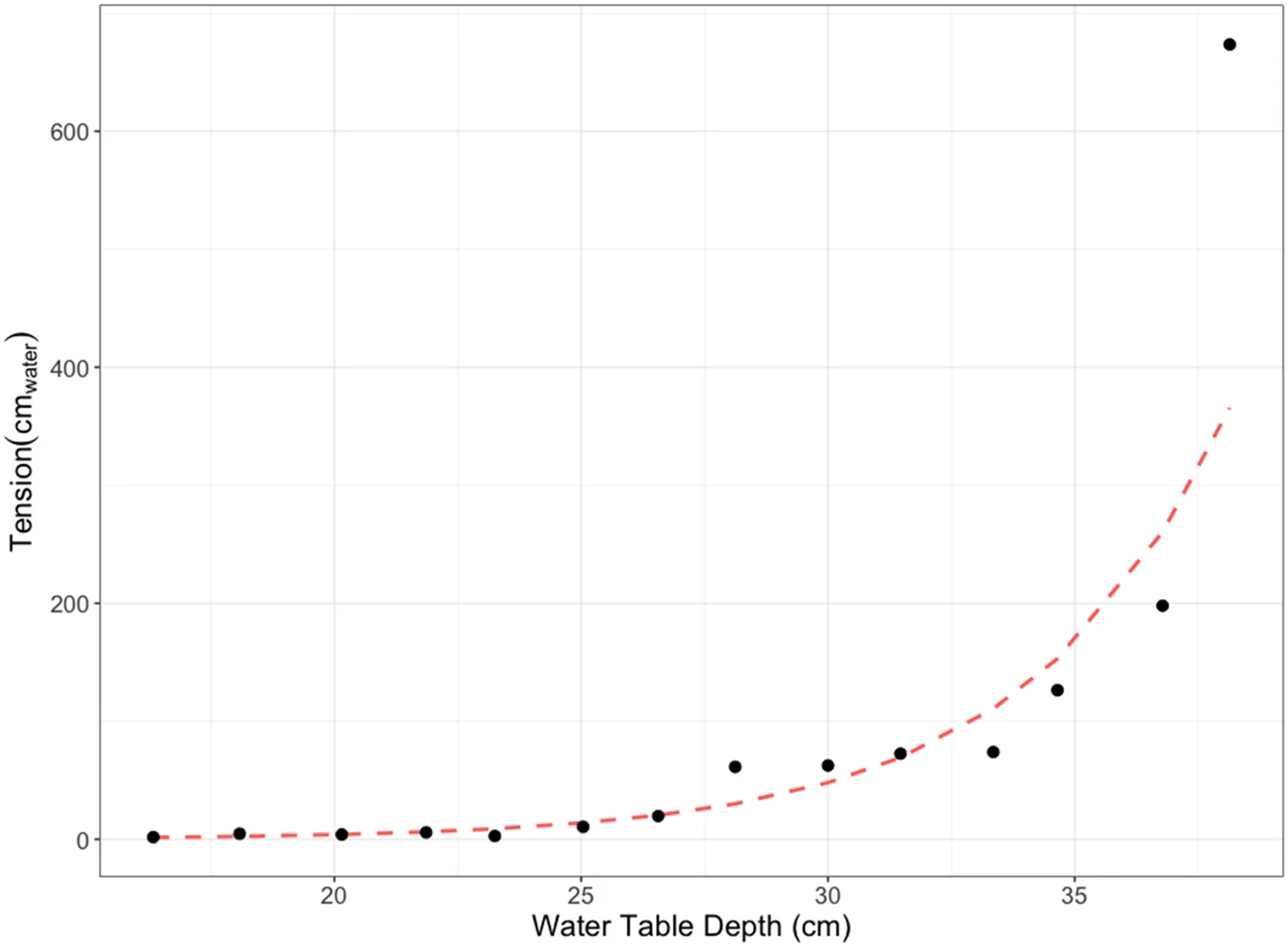
Near-surface tension and water table depth, both in cm (of water equivalents for tension). Data from a laboratory study using a growth chamber set to 40% humidity with circulation fans. Tension measured using staggered mini-tensiometers within peat samples [33].

Using static values for aerodynamic resistance (83 s m^-1^), saturated vapour density (0.017 kg m^-3^), and relative humidity (0.0068 kg m^-3^) we developed the following equation to describe the limitation of AET (with respect to PET) with increasing water table depth (WTD) (Fig. 6, Eq. 11). This corresponds well to the observed “threshold” effect of water table (WT) – surface hydrologic disconnection at a 40 cm WTD [34].

**Fig. 6:**
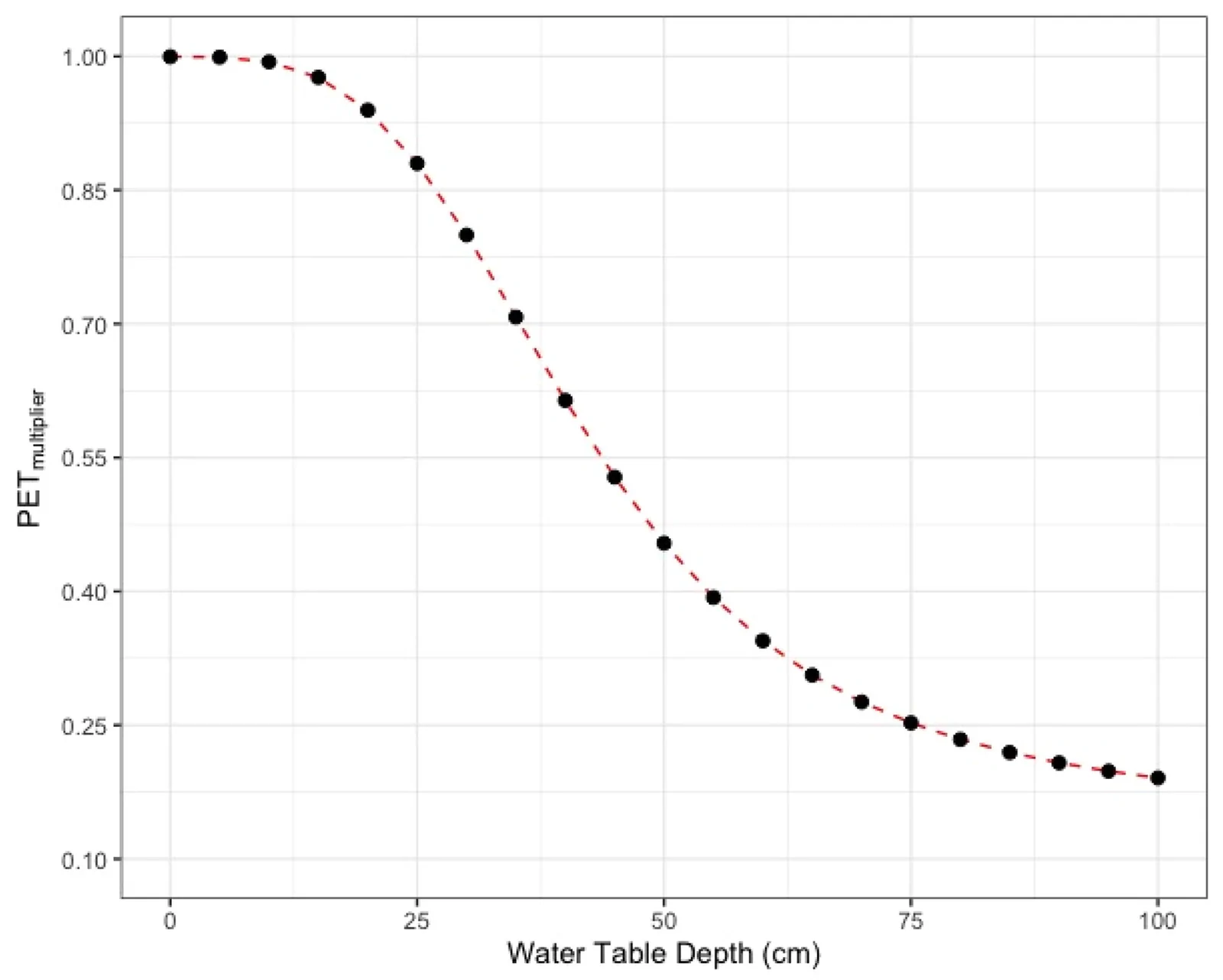
PET_multiplier_ (PET to AET function) when the water table is provided in cm and the AET to PET ratio is limited to 0.15 by setting C = 0.15. Note that water table depth is substituted for the Peat Moisture Code during calculation.

Note that throughout the exemplary PMC equation, the PET to AET function (*PET_multiplier_*) is limited to a minimum of 0.15 (*i.e*., C = 0.15) to account for ET from trees and shrubs continuing even after surface evaporation has shut down [32] (Fig. 6).

Equation for PET to AET when WTD is in cm, and the PET_multiplier_ is limited to 0.15:

(11) $$PET_{multiplier}=1-\left(\frac{0.0004+\left(1-C\right)}{1+e^{-3.45*\left(log\;\left(WTD\right)\;-3.743\right)}}\right)$$

Note: *C* can be adjusted to strengthen or weaken the PET to AET function. Generally, smaller values of *C* (*e.g.*, 0.1) represent a steeper gradient with increasing WTD, whereas higher values of *C* (*e.g.,* 0.3) represent a shallower line as the *PET_multiplier_* is limited by *C*. Future local calibration across different peatland types could modify this value, but it is fixed at 0.15 for standard calculations.

### Effective precipitation (Eff_Precip_)

Actual Effective precipitation (*Eff_Precip_*) represents the amount of water being added to the peatland system daily. This is entered into the PMC calculation as a daily sum and can be from measured or modelled data sources. If measured data contain gaps these should be filled with estimates from modelled sources such as satellite products. Depending on the source, precipitation data may represent rain only, or rain and snow as total precipitation (cm of water equivalent); both forms can be accounted for in the PMC (Fig. 7). There is an option to set a minimum amount of precipitation required before water reaches the peatland storage (and therefore affects the PMC). This amount can be set by the user. The general PMC, here, is parameterised to a treed bog with ∼ 60% canopy closure predominantly made up of stunted black spruce trees. For this system, we set a daily precipitation value of 1 mm (0.1 cm), effectively converting all daily precipitation values less than 1 mm to 0 and keeping all daily precipitation values above 1 mm unchanged (Fig. 8). This is a lower rainfall interception compared to the 1.5 mm in the Duff Moisture Code (DMC), owing to the reduced canopy cover in treed peatlands compared to the reference pine stand in the DMC.

**Fig. 7:**
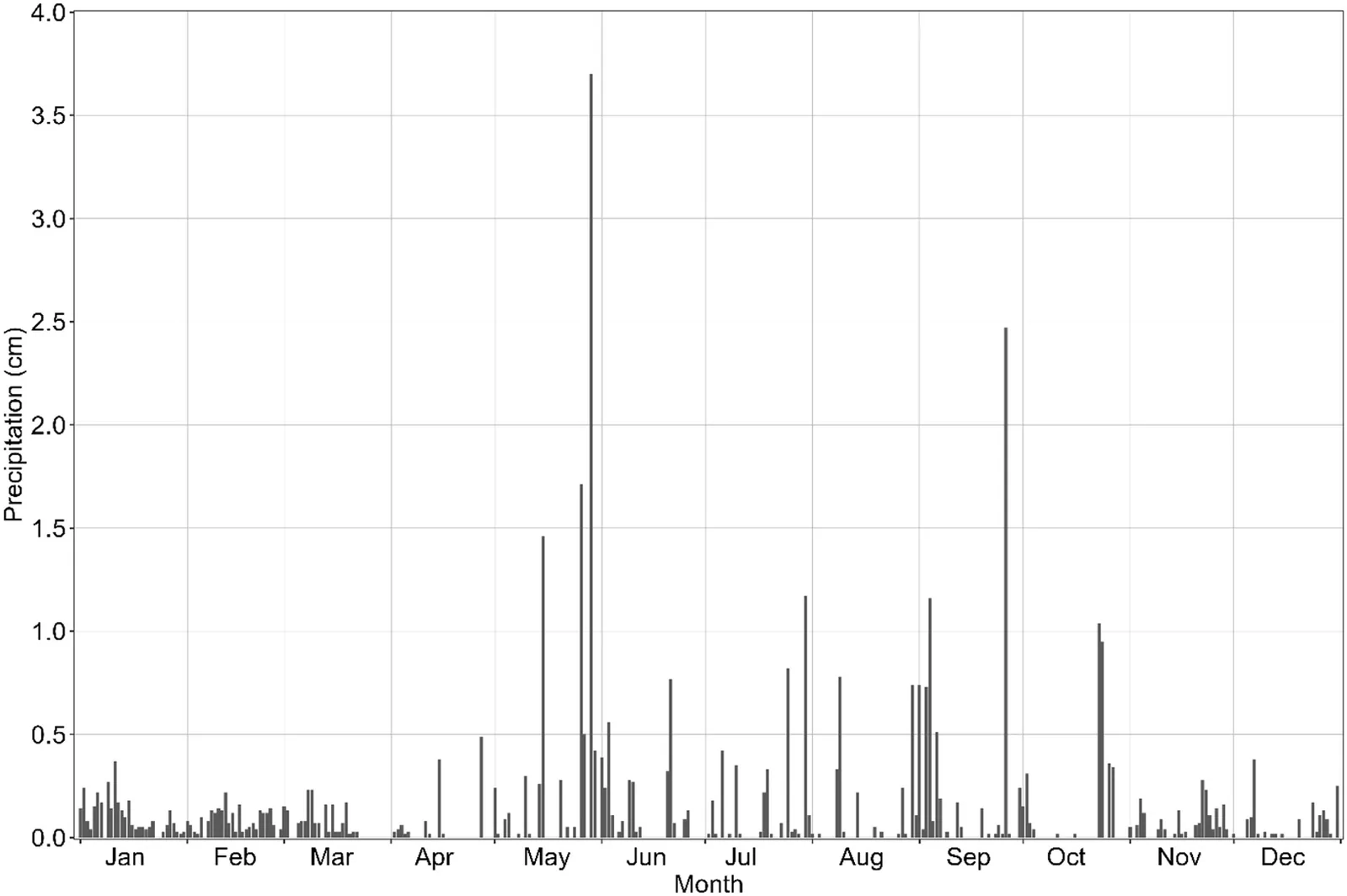
Example of daily summed precipitation (rain and snow, in water equivalent) from Mildred Lake, AB.

**Fig. 8:**
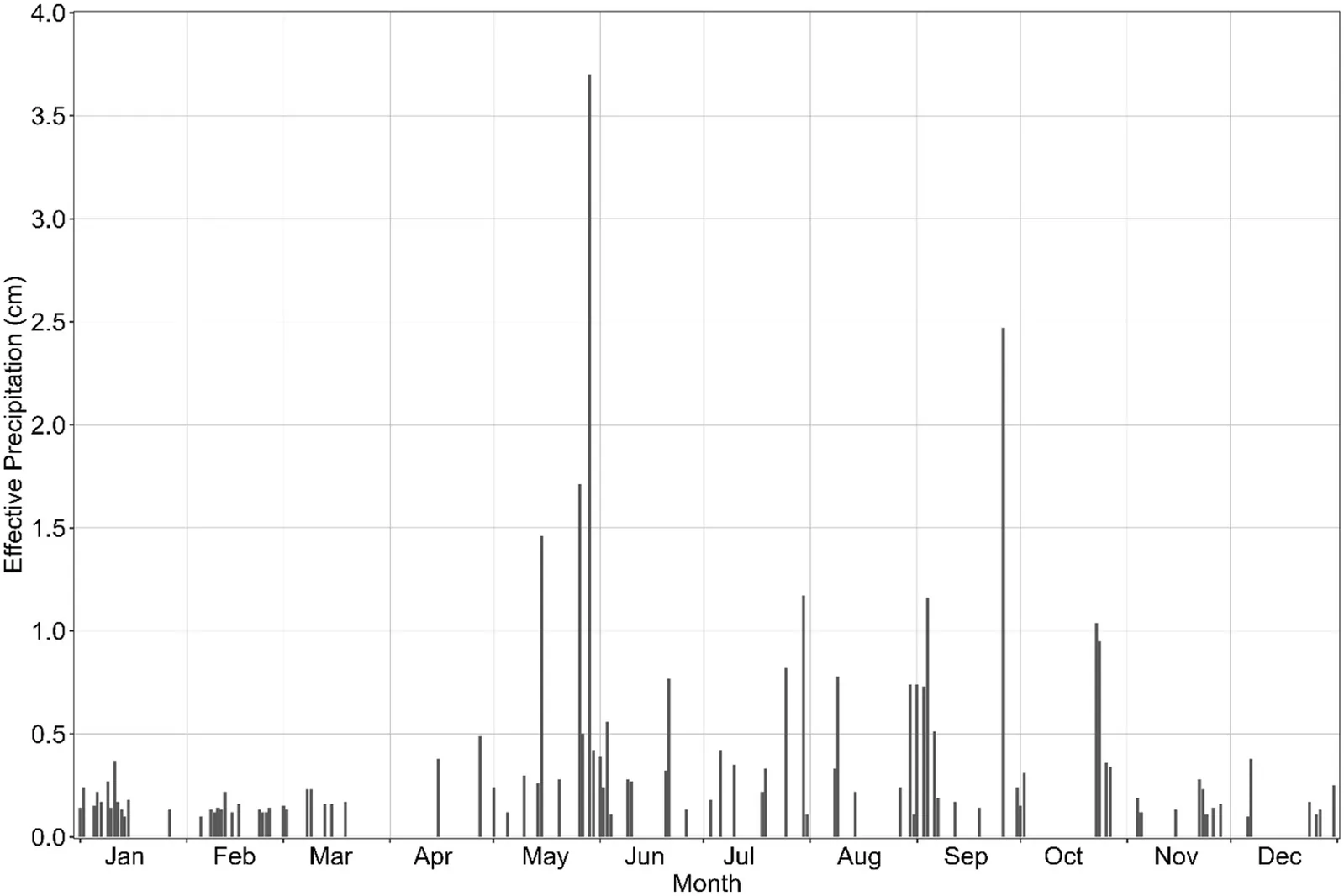
Example of daily summed precipitation (rain and snow, in water equivalent) from Mildred Lake, AB, using a 1 mm (0.1 cm) interception threshold to calculate effective precipitation, i.e., that which enters the system. In the default daily Peat Moisture Code (PMC) calculation, all interception from daily totals greater than 1 mm enters the system.

Given that *Eff_Precip_* represents the only input of water into the system, the value could also be adjusted to account for consistent surface water or groundwater input. In this default example, we do not include surface or ground water input (or output) as the system modelled is a treed bog (precipitation-fed). Furthermore, *Eff_Precip_* can be used to overwinter the PMC in cases where year-round data is not available, or snow as precipitation is not available, by attributing a portion of the maximum snow water equivalent to the system. In this example, no modifications to the overwintering of the PMC were made to account for the effect of ground ice and limited recharge of snow into peatland storage [35] or ablation. However, a simple constant reduction in winter-time precipitation could be made to account for the above processes. In mineral soil deposits and areas with seasonal ground ice presence, we suggest a default reduction of 10% for winter-time precipitation effectiveness (in addition to interception reductions).

### Specific yield (Sy)

Specific yield (*Sy*) is used to describe the sensitivity of the PMC to water inputs and outputs. *Sy* is a ratio between 0 and 1, which indicates the amount of water released due to drainage from lowering the WT in an unconfined aquifer [36]. It is often estimated as the difference between saturated (volumetric) moisture content and the moisture content at free drainage or a very low soil water tension (*e.g*., 5 hPa) for soil samples. In Peatland hydrology, *Sy* can be interpreted as the ratio of the movement of the WT for the addition or removal of water from the system. In this way, a *Sy* value of 0.1 would mean that the WT moves 10 times the depth of the water added or removed through precipitation or evapotranspiration; for example, when *Sy* is 0.1, every 1 mm of rainfall added to the peat raises the WT by 10 mm.

*Sy* is dependent on the pore structure of peats and tends to decrease (towards 0) with increasing peat decomposition and associated increasing bulk density [30]. As with bulk density, *Sy* tends to change with depth in a non-linear fashion. Based on a set of samples of peat from hollows in bogs and treed poor fens across boreal Alberta [30], an exponential curve was determined to best describe this relationship (Fig. 9; Eq. 12; R^2^ = 0.91)

(12) $$Sy=A*e^{\left(-B*PMC_{i-1}\right)}$$

**Fig. 9:**
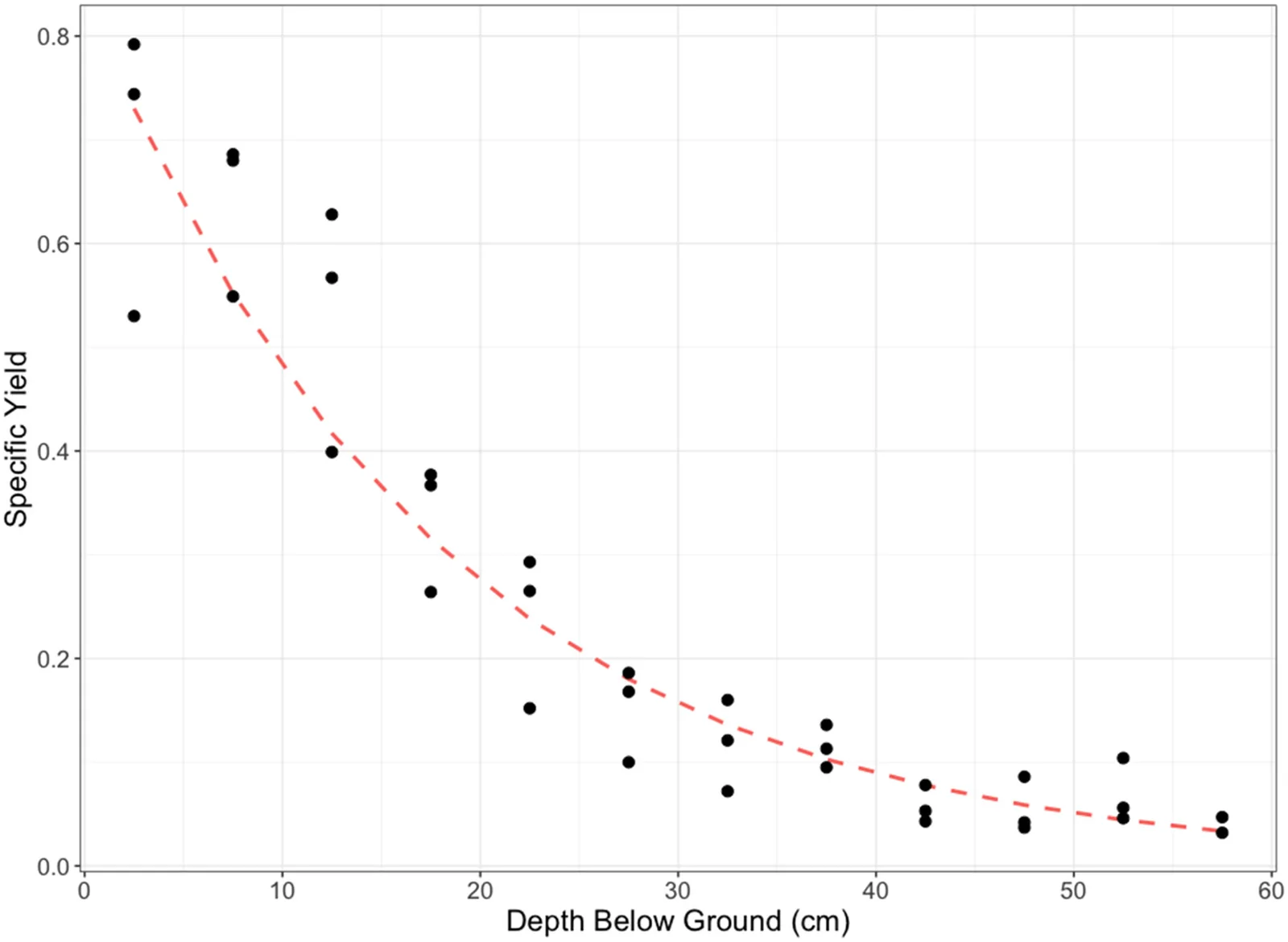
Change in specific yield with depth below ground for a set of samples from hollows in treed poor fens across boreal Alberta. The fitted exponential relationship is used to parameterise the change in Sy with depth (Eq. 12).

Where, A = 0.8674, B = 0.0540 and i = day, and therefore PMC_i-1_ represents the estimated WTD on the previous day. Note that *Sy* is limited to a minimum value of 0.1 in the PMC equation.

The exponential function of *Sy* decline with WTD can be fitted using other sets of peat samples. Thus, it could theoretically be calibrated to a particular region or peatland type, for example, shallower peatlands in a Boreal Shield rock barrens landscape, and drained or harvested peatlands.

The *Sy* (1/*Sy*) is applied to the net change in storage ($Eff_{Precip_{i}}$ - AET_i_) to determine the change in PMC. A minimum *Sy* of 0.1 (maximum multiplier of 10) has been adopted for the general PMC calculation to negate exceptionally large changes in storage at high PMC values. In some cases, where the PMC value of the previous day is high (and thus the *Sy* value used is quite low) and a large rain event occurs, adding a high input of precipitation into the model in a single day, this simplification of the *Sy* and WT rise relationship can result in erroneously high changes (decreases) in the PMC. In reality, as the WT rises through the peat profile, it will continuously encounter peat of higher and higher *Sy,* and thus the effective change in the WTD for each unit of precipitation added will diminish. A user may adapt this component of the PMC to reduce the PMC change under specific conditions. For example, by setting a daily precipitation threshold amount (*e.g.*, 20 mm) and applying a specific *Sy* value (*e.g.*, 0.3; notably higher than the potential actual *Sy* deep within the profile, which could be as low as 0.1) to represent the average *Sy* of the peat that will be encountered as the WT rises through the profile. For the example calculations provided, this was not deemed necessary, but regions with sporadic rainfall may require such a modification.

The Peat Moisture Code (PMC)

A daily bookkeeping approach is used to calculate the PMC as follows:

(13) $$PMC_{i}=PMC_{i-1}+\left(\left(AET_{i}-Ef{f_{Precip}}_{i}\right)\;/\;Sy\right)$$

Where *PMC_i_* is the PMC value of a given day [i], *PMC_i-1_* is the PMC of the previous day (or the chosen start-up PMC value if i = 1), *AET_i_* and $Eff_{Precip_{i}}$ are the AET and effective precipitation of the given day, and *Sy* is the *Sy* based on the value *PMC_i-1_* (Fig. 10)

**Fig. 10:**
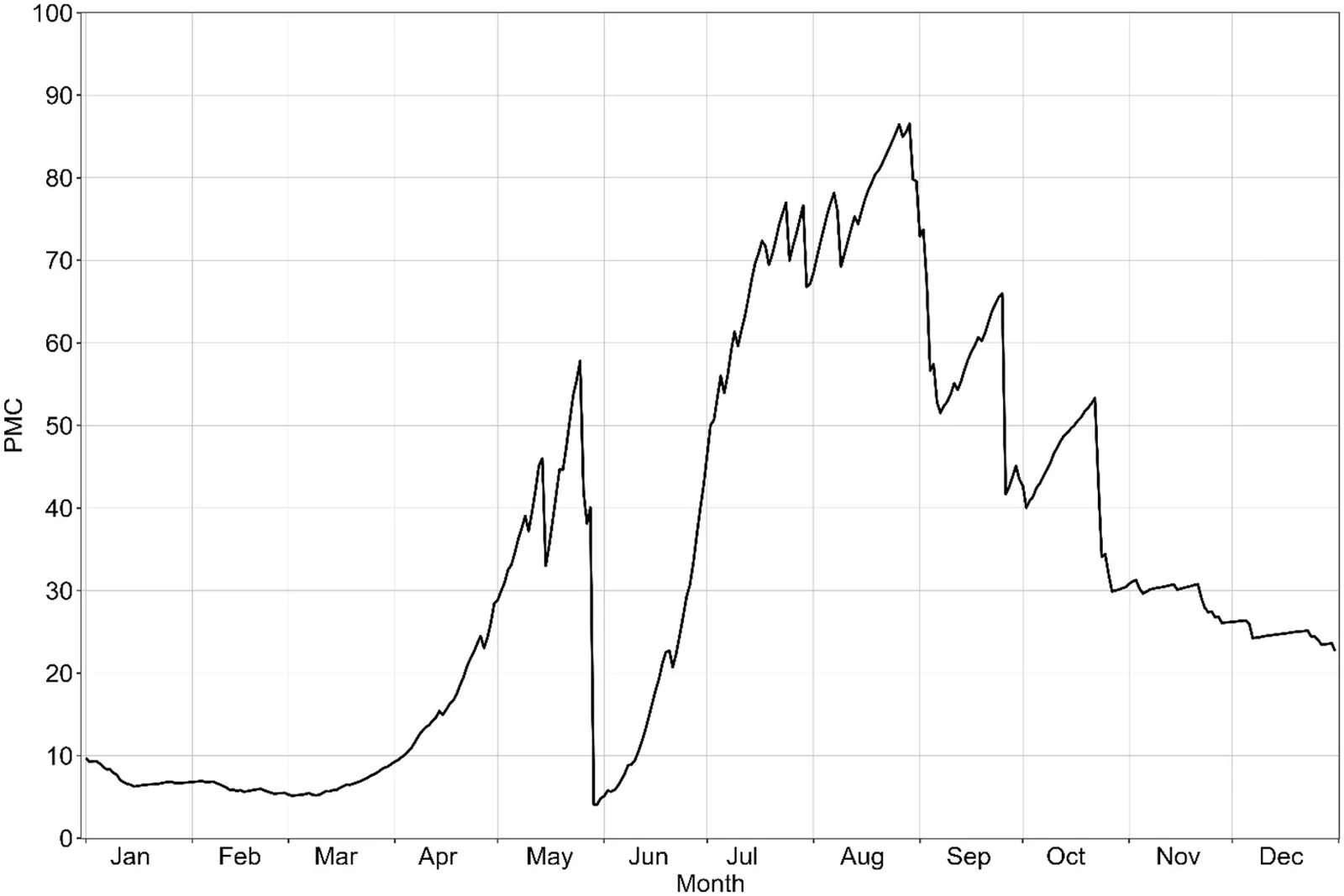
The Peat Moisture Code (PMC) over one year for Mildred Lake, AB, calculated with modelled potential evapotranspiration (PET) and effective precipitation (Eff_Precip_). Note that the decrease in PMC at the end of May corresponds to a series of large effective precipitation events shown in Fig. 8.

All of the above equations are compiled together in the PMC R package, hosted on GitHub, which is listed in the Specifications Table. Users are able to modify the code as they see fit, relating to their specific ecosystem type or data availability. This method forms the basis of the hourly PMC that will be integrated into the FWI system of the CFFDRS. The hourly PMC method will be documented in a technical report published by Natural Resources Canada; this hourly PMC will not be modifiable by users but will serve as a consistent method for assessing peatland fire danger across the country. Once integrated, it will be calculated at all federal weather stations and available publicly.

### Application and interpretation

The PMC has been developed to address the growing need to quantify, analyze, and forecast fire danger associated with belowground peat and aboveground peatland fuels. As the code is implemented in varying ecozones and for a variety of peatland types, ecozone- or region-specific thresholds to fire danger will be derived. At present, the suggested thresholds are qualitative in nature, but as the daily PMC is back-calculated and analyzed, different thresholds will be associated with physically-based fire metrics (*e.g.*, rate of spread, depth of burn, ignition success and/or % burned) to give more context and provide real-world interpretation of the fire danger thresholds presented below. Based on the current understanding and available data, this section describes some critical periods of peatland fire risk and how the PMC can be used to identify them.

### Seasonal fire potential

For most provinces and territories across Canada, the fire season begins in May and runs through September [37], while in British Columbia (BC), the fire season tends to be concentrated in July and August. However, climate change is driving earlier fire season starts and later finishes in BC [38,39] and the rest of Canada [40]. Mega fires (fires which burn >200,000 ha) are increasing in occurrence, and fire size is increasing in most regions [41], as are conditions conducive for high-severity fire [42]. A typical annual hydrological cycle for peatland-dominated areas in the northern hemisphere will consist of a spring recharge period (February – April) driven by snow melt and rain, followed by a prolonged or punctuated series of drying events throughout the late spring and summer (May – September), before rewetting occurs heading into the fall and winter (October – January). Critically, many regions across Canada experience seasonal ground ice that may act to limit moisture transport to the evaporating surface, promoting dry surface fuels despite (relatively) low PMC values. To account for this process, we suggest two sets of fire danger category thresholds based on the timing of the so-called “ice-out” (the date on which the majority of seasonal ground ice has thawed; Fig. 11). Before region-specific analyses are completed, we suggest that the following PMC values will provide reasonable guidance to different levels of peatland fire danger (Tables 5 & 6).

**Fig. 11:**
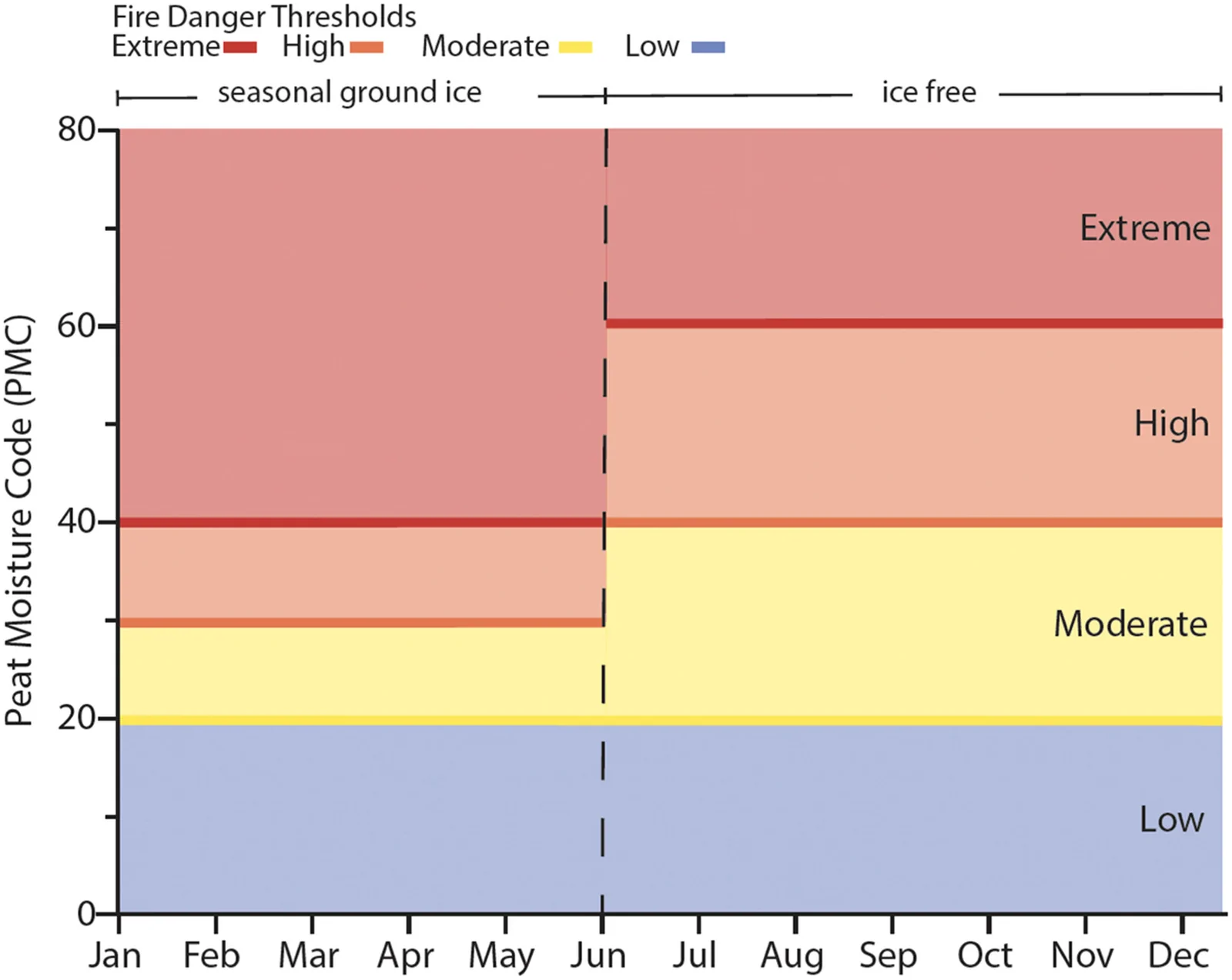
Peat Moisture Code fire danger threshold values over a year showing time-of-year dependent thresholds.

**Table 5:** Daily Peat Moisture Code (PMC) values and corresponding fire danger categories for seasonal ground ice conditions or in areas of permafrost.

*PMC Range*	*Fire Danger Categorization (With ice present)*
-10 to 0	No smouldering fire danger
0 to 20	Little to no smouldering fire danger
20 to 30	Moderate smouldering fire danger
30 to 40	High smouldering fire danger
40+	Extreme smouldering fire danger

**Table 6:** Daily Peat Moisture Code (PMC) values and corresponding fire danger categories for Summer to Fall conditions or in areas with little to no presence of seasonal ground ice or permafrost.

*PMC Range*	*Fire Danger Categorization (With no ice present)*
-10 to 0	No smouldering fire danger
0 to 20	Little to no smouldering fire danger
20 to 40	Moderate smouldering fire danger
40 to 60	High smouldering fire danger
60+	Extreme smouldering fire danger

### Early-season fire potential

While some areas in Canada, especially in southern and/or eastern regions, do not develop seasonal ground ice and are not impacted by permafrost (Fig. 12A and B), areas that experience low winter temperatures and relatively shallow snowpacks tend to develop seasonal ground ice, especially in soils with high water content (*e.g.*, peat). This frozen ground influences fire risk in peatlands [18,43,44]. If the peat is at (or near) saturation at the time of freezing, the ground ice forms as “concrete” lenses (Fig. 12C). Concrete ice lenses affect the water balance (and fire potential) in peatlands in two main ways. Firstly, ice can inhibit the infiltration of water from snowmelt into peatlands [35,45], reducing the total amount of spring recharge. Secondly, ice can act to block the upwards transport of moisture from the WT (if it is below the ice level) to the evaporating surface of the peat and cause the upper layers of the peat to dry rapidly [46]. These processes can lead to low surface moisture conditions and high fire potential in the early fire season, before ice-out (Fig. 12C), similar to when WT positions are very deep, such as in mid-summer (Fig. 12B).

**Fig. 12:**
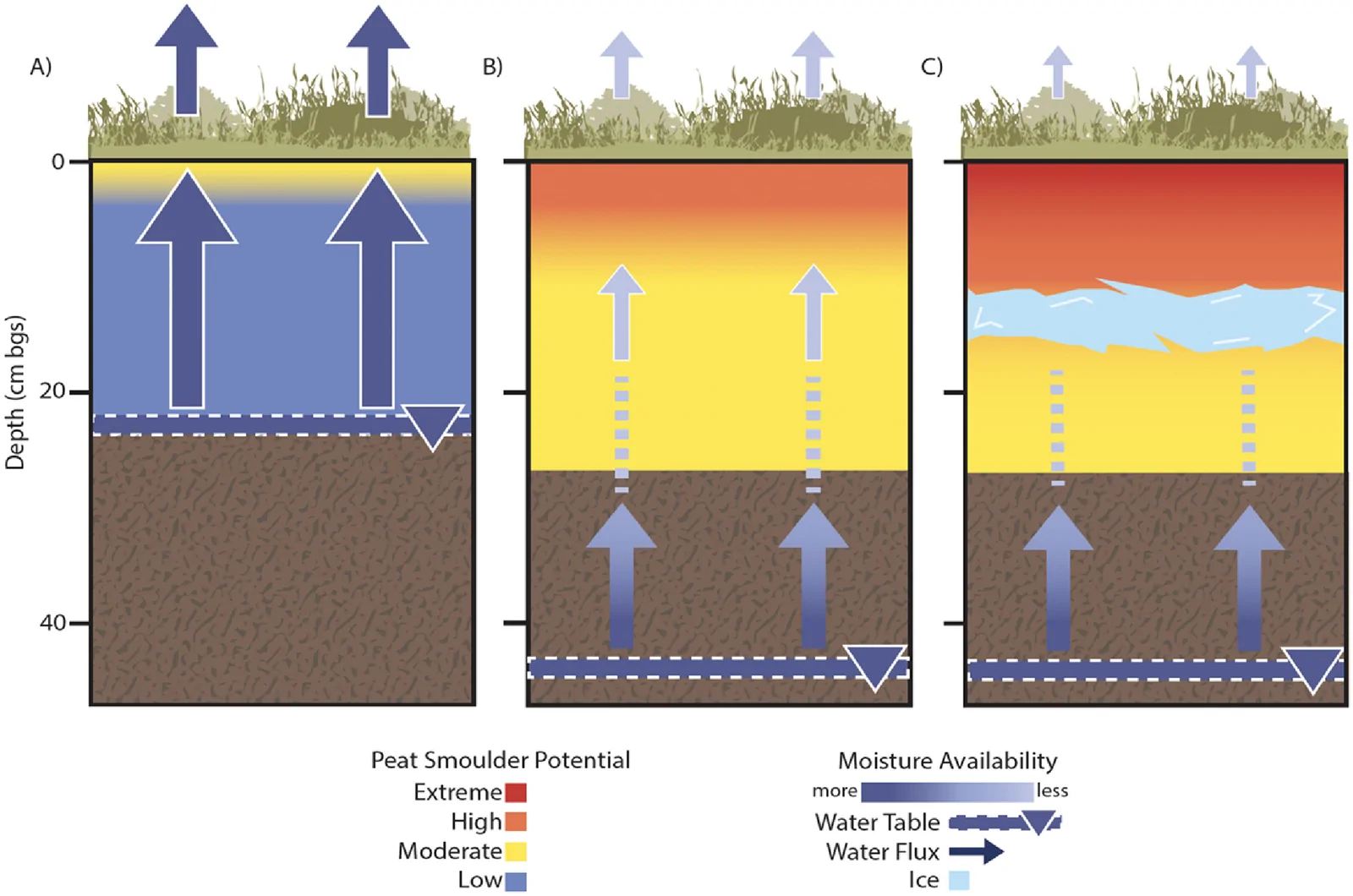
The impact of seasonal ground ice (or permafrost) and water table (WT) position on peat smouldering potential. A) No ice - uninhibited evaporation from peat surface with supply from WT, typically associated with ice-free regions or post ice-out. B) Low (>40 cm deep) WT position leading to limited water supply to the surface and moderate to high peat smouldering potential in the near surface, typically associated with periods of drying or drought. C) Ice lens (or permafrost) blocks upward transport of water from the WT, leading to high (to extreme) smouldering potential above the ice, typically associated with spring conditions across the boreal or year-round conditions in permafrost regions. Blue arrows denote movement of water within the peat profile, and to the atmosphere via evaporation, with larger size and darker colour denoting more water movement.

Similarly, permafrost peatlands are likely to experience these conditions year-round due to the continuous presence of frozen ground. Low surface moisture conditions and/or rapid drying in the early season can drastically enhance fire potential in peatlands with ice present (Fig. 12). To account for this, we suggest an interpretation which uses lower thresholds for peatland fire danger in the early season (Fig. 11), especially in areas with significant seasonal ground ice or with permafrost present.

### Mid-season fire potential

The PMC is expected to follow a typical seasonal trend where evapotranspiration peaks in summer (June to September), corresponding to drier peat and peatland vegetation. This typical seasonal variation (as described in *Seasonal fire potential*) is built into the categorization of the PMC fire danger values, where, in ice-free regions or following ice-out in seasonal ground ice regions, water can flow effectively to the surface of the peatland (Fig. 12A) until a critical WTD is reached (Fig. 12B). Hence, fire danger thresholds for ice free conditions (Fig. 13; Table 6) are comparably higher than those in the presence of ground ice (Fig. 13; Table 5).

**Fig. 13:**
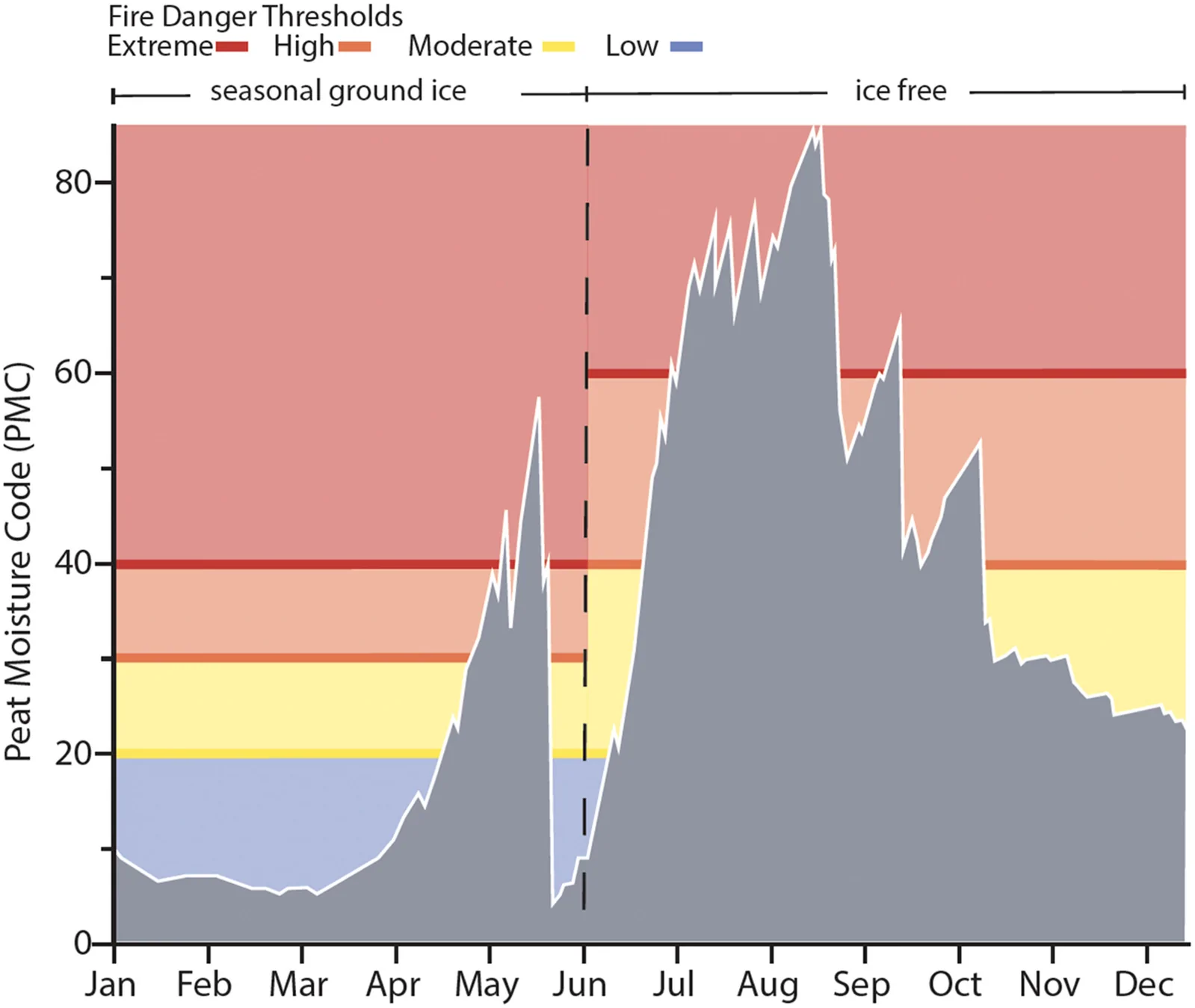
The Peat Moisture Code (PMC) over one year, showing a rapid and early increase in early-season (spring) fire danger, followed by a decrease in PMC associated with rainfall before further increase throughout the summer. Note the lower thresholds for fire danger thresholds in months with seasonal ground ice presence (Jan-Jun).

### Continued development of the PMC

In general, the greatest fire potential in peatlands occurs when above- and below-ground fuel moisture are coupled and low. In these scenarios, fire can spread rapidly across the surface or through the canopy and can also propagate into the peat layers below ground, causing both fire spread and mop-up challenges. High PMC values in the fall may suggest increased risk of overwintering (smouldering) fires that represent a risk of transitioning back into flaming fires, reinitiating direct threats of fire on communities or values [12]. Further, prolonged smouldering can cause chronic air quality, water quality and visibility issues for people, livestock, industry and transportation [47]. As fire managers interact with the PMC in their own region and in different ecosystem types, we expect time- and place-specific interpretations to naturally develop.

The continued development of the daily PMC, the CFFDRS hourly PMC, along with the development of peatland fuel types in the CFFDRS Fire Behaviour Prediction (FBP) system, will enhance our ability to assess, predict, and respond to deep organic soil and peatland fire risk. As historic values are back-calculated to assist with benchmarking current values and trends, and as the PMC is measured and recorded at local to regional scales, typical patterns, departures from typical patterns and links to real-world fire metrics (number of ignitions, peatland area burned) will be possible. As multi-year datasets are developed, specific fire danger features can be identified, such as those shown in Fig. 14, including a large high-severity wildfire (PS33; 11,000 ha) in a peatland-dominated region in north central Ontario that was ignited on July 18^th^, 2018, and smouldered in peat for many months following ignition.

**Fig. 14:**
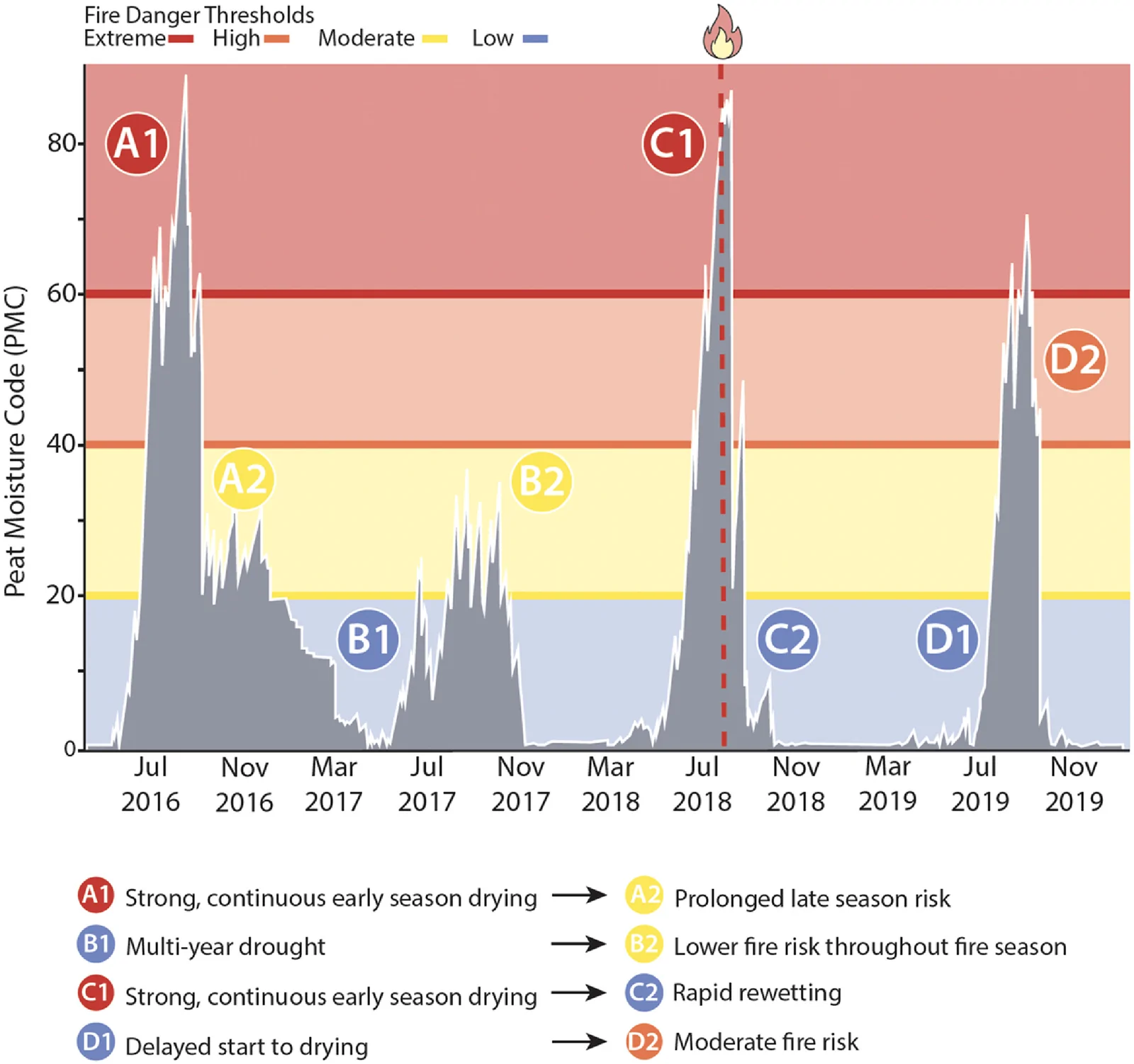
A multi-year dataset of the Peat Moisture Code (PMC) calculated using on-site weather data from a peatland-dominated region near Nobel, Ontario, in the Boreal Shield ecozone. Key fire danger features shown by the PMC are highlighted and described by letters/numbers throughout the period.

## Method validation

We have developed the daily PMC to track the moisture status in peatland fuels. It is expected to loosely approximate the WTD in a treed bog (peatland). Evaluation over multiple years provides information regarding the potential fire danger of peatland fuels. The PMC increases as WTD increases (below ground), which corresponds to an increase in fire danger. In this method, parameterisation of functions was made using data from field evaluation (*e.g*., Priestley-Taylor alpha; Table 4) and laboratory experiments (*e.g*., surface tension; Eq. 10; specific yield; Eq. 12). Below, we compare the daily PMC with the WTD from two peatlands in north central Ontario, Canada (Fig. 15). The goal of the PMC is to show weekly to monthly trends in fire danger, while also providing an evaluation of multi-year drought. As such, we used qualitative analysis to determine the robustness of the daily PMC for these purposes. The figure below (Fig. 16) shows the same data as Fig. 15 with the addition of WTD data from nearby peatland sites and daily summed precipitation data.

**Fig. 15:**
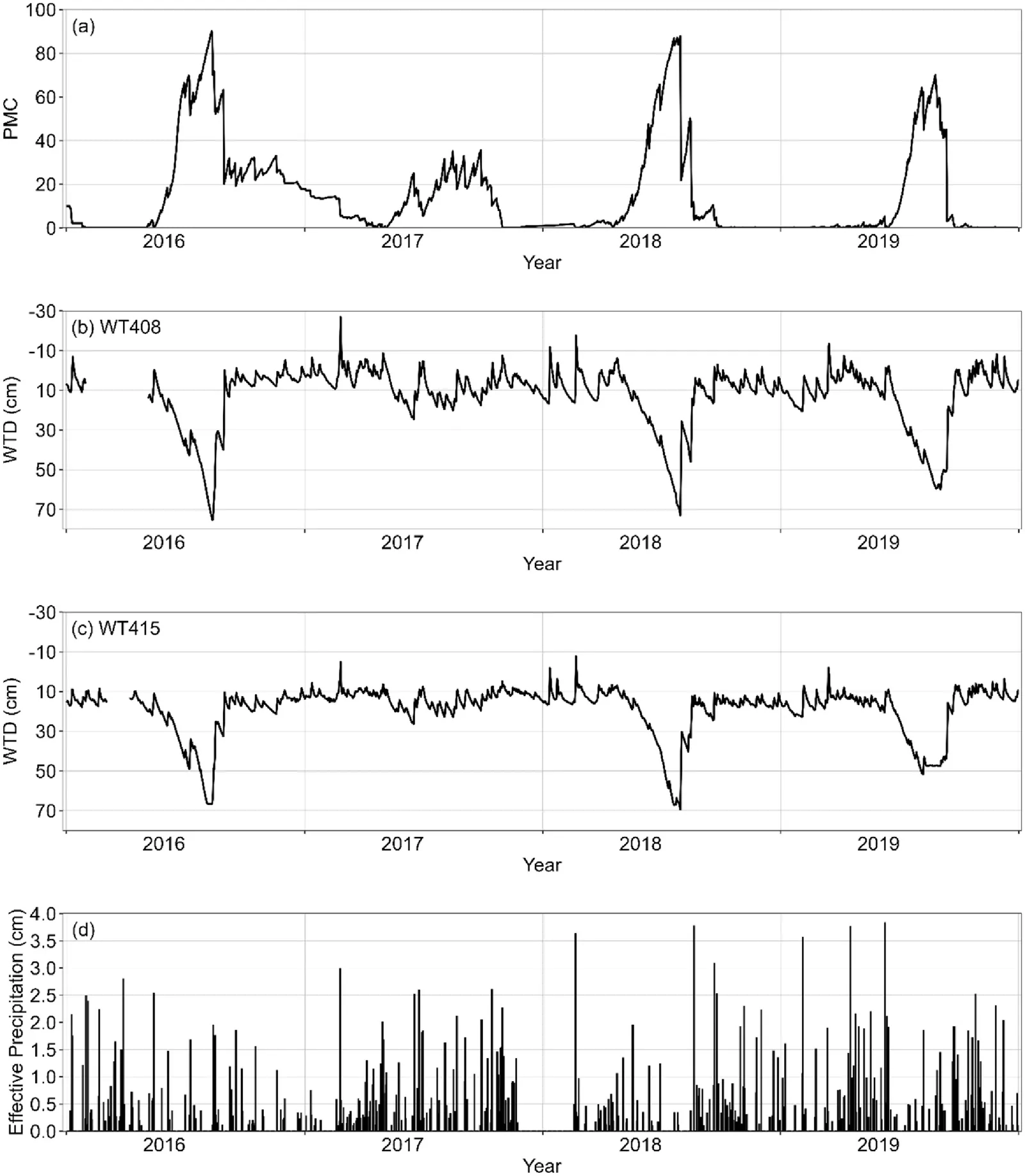
a) The Peat Moisture Code (PMC) over a four-year period calculated from on-site weather data in a peatland-rock barrens landscape near Nobel, Ontario. b) Continuous water table depth (cm) measurements from a small peatland in the region. c) Continuous water table depth (cm) measurements from a larger peatland in the region. Note that the missing data in July 2018 was due to a large (11,000 ha) fire in the area (PS33). d) Effective Precipitation (cm) input into the PMC model.

**Fig. 16:**
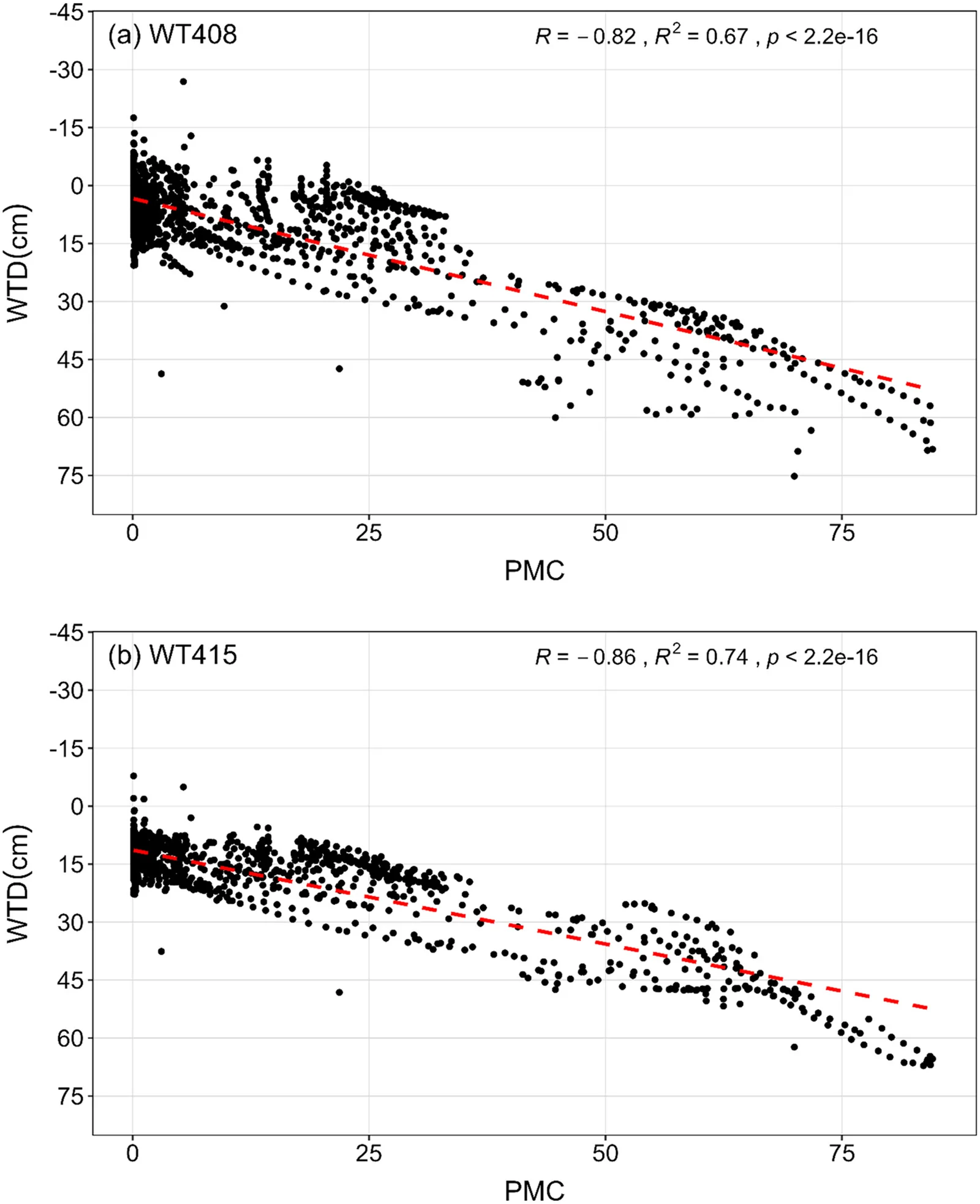
Correlations between the daily Peat Moisture Code and the Water Table Depth (WTD) for (a) peatland site 408 and (b) peatland site 415, near Nobel, Ontario.

The daily PMC captures year-over-year differences in fire danger, where, in the region of interest, 2016 and 2018 were relatively hot, dry years, and 2017 was a wet year [48]. Within-season variability in fire danger is also captured well as WT drawdown periods (increasing depth belowground) correspond to increasing PMC values with a similar timing and magnitude. Decreases in PMC value (and increases in WT position) are driven by large or consecutive precipitation events. A large (11,000 ha) fire occurred in the region, which is dominated by peatlands and unproductive forests, and burned through mid to late July 2018 when the back-calculated PMC shows Extreme Fire Danger with a value of ∼80. The fire was both high intensity and high severity and burned for months in peatland organic soils before being completely extinguished [24].

## Limitations

The limitations of the daily PMC can be grouped into the following themes: 1) Reliance on field samples largely from one region, 2) Simplifying complex ecohydrological processes into unified equations, and 3) Subjectivity of the interpretation of code values.

This method uses equations and variables that are parametrised from field data and field samples processed in the laboratory. Most of the field data and samples are from boreal Alberta in western Canada, specifically from treed bogs and treed poor fen peatlands. While different peatland types and peatlands across different regions have similar structure and functions, peatland type can change the strength of some feedbacks that may affect moisture retention [13]. As a test of the transferability of the method, we tested the PMC against WTD values from two poor fens in the Boreal Shield Ontario in eastern Canada, and we found that the code was robust.

To ensure uptake of the method by varied users and to reduce computational expense, we simplified some of the water input and output processes into simple, unified equations, sometimes using pre-existing default parameters, such as the Priestley-Taylor alpha coefficient. Notably, the limitation of actual evapotranspiration in relation to potential evapotranspiration is presented with one equation, despite it being based on a number of complex processes. However, care was taken to preserve the fundamental physics of these processes. Similarly, the interception scheme is simple and is applied regardless of precipitation type (*i.e.*, rain, snow, mix). While we acknowledge that interception will differ based on precipitation type, we aimed to track the general trends and status of moisture in peatland fuels, and this simplification, at this time, doesn’t appear to affect that outcome. Different interception regimes will produce slightly different magnitudes of PMC values, but trends will remain similar to the default shown here.

Lastly, the daily PMC, as presented here, is designed to be flexible and applicable to a wide range of peatland types and ecoregions. The average and range of PMC values will likely differ across the country (or in countries other than Canada), and care should be taken to understand the typical range and trends in PMC before interpreting results. PMC values are not yet associated with specific real-world fire implications, such as probability of successful ignition in peatlands, or peatland area burned or burn severity; however, as the daily and hourly PMC methods are expanded for use across Canada, these metrics will be developed and shared with the scientific, industrial, and fire management communities.

## Ethics statements

Not applicable.

## Related research article

None

## Supplementary material *and/or* additional information

None

## CRediT authorship contribution statement

**S.L. Wilkinson:** Conceptualization, Methodology, Writing – original draft, Formal analysis, Visualization, Investigation, Writing – review & editing. **G.J. Verkaik:** Conceptualization, Methodology, Writing – original draft, Writing – review & editing. **D.K. Thompson:** Conceptualization, Methodology, Writing – original draft. **J.M. Waddington:** Conceptualization, Methodology, Writing – original draft, Writing – review & editing, Funding acquisition. **E. Arango Ruda:** Visualization. **B.M. Wotton:** Conceptualization, Methodology, Writing – original draft, Funding acquisition.

## Declaration of competing interest

The authors declare that they have no known competing financial interests or personal relationships that could have appeared to influence the work reported in this paper.

## Data Availability

The GitHub repository () contains the PMC R package with a compilation of functions and with the equation to calculate the daily Peat Moisture Code, as well as four climate datasets used in this paper from ClimateData.ca.https://github.com/ElizabethArangoRuda/PMC
